# Supplementary material for: The genome sequence of a ground beetle, Dromius quadrimaculatus (Linnaeus, 1758) (Coleoptera: Carabidae)
Source: Wellcome Open Res. 2025 Oct 6;10:555. [Version 1] doi: 10.12688/wellcomeopenres.24898.1 (PMC12759285; doi:10.12688/wellcomeopenres.24898.1)
Supplement: Peer review history

# Reviewer report: Reviewer response for version 1

**DOI:** 10.21956/wellcomeopenres.27425.r142545

**Published:** 2026-01-05

**Authors:** Nadège Guiglielmoni

**Affiliations:** 1 Universite Libre de Bruxelles Departement de Biologie des Organismes (Ringgold ID: 168978), Brussels, Brussels, Belgium; 2 University of Cologne Center for Molecular Medicine Cologne, Cologne, Germany

This study presents a chromosome-level genome assembly of a female individual of the arboreal carabid species *Dromius quadrimaculatus. *PacBio HiFi and Hi-C reads, from two different individuals, were combined to produce chromosome candidates using hifiasm and YaHS, and MitoHiFi for the mitochondrial genome. Genome evaluation supports correctness and completeness of the assembly.

 I have a few small modifications to suggest:

 1) I would spell out that the species has XY chromosomes.

 2) I would detail for all the sequencing datasets the sex of the specimen, especially for those who may want to reuse them.

 3) I am confused why this plot from Merqury was incorporated with pri/alt/shared. If you used the primary assembly, I would expect to see the spectra-cn plot of the primary. And is the QV indeed for the primary of pri+alt?

Are sufficient details of methods and materials provided to allow replication by others?

Yes

Is the rationale for creating the dataset(s) clearly described?

Yes

Are the datasets clearly presented in a useable and accessible format?

Yes

Are the protocols appropriate and is the work technically sound?

Yes

Reviewer Expertise:

Genome assembly, animal genomics, animal parthenogenesis

I confirm that I have read this submission and believe that I have an appropriate level of expertise to confirm that it is of an acceptable scientific standard, however I have significant reservations, as outlined above.

# Reviewer report: Reviewer response for version 1

**DOI:** 10.21956/wellcomeopenres.27425.r142548

**Published:** 2026-01-02

**Authors:** Liang Lu

**Affiliations:** 1 Hebei Normal University, Shijiazhuang, Hebei, China

I have read the main text of the manuscript, which reports a very commonly found (in the UK) ground beetle, Dromius quadrimaculatus (Linnaeus, 1758). It is a formulaic paper that reports an NGS sequence with detailed description of the background of the sample information in taxonomy, biology, collection, etc. The authors kindly presented in this manuscript clear images that make their identification trustable. The workflows of sample preparation, DNA extraction, sequencing, and assembly are fine and aligned with previous work/publications by Wellcome Sanger Institute. But why this time they do not include annotation? So I suggest the author should provide details of prediction and annotation, methods, results (with the assembly), and a report on the results.

Are sufficient details of methods and materials provided to allow replication by others?

Yes

Is the rationale for creating the dataset(s) clearly described?

Yes

Are the datasets clearly presented in a useable and accessible format?

Yes

Are the protocols appropriate and is the work technically sound?

Yes

Reviewer Expertise:

Zoology (Coleoptera), Phylogenomics

I confirm that I have read this submission and believe that I have an appropriate level of expertise to confirm that it is of an acceptable scientific standard.

# Reviewer report: Reviewer response for version 1

**DOI:** 10.21956/wellcomeopenres.27425.r137098

**Published:** 2025-11-12

**Authors:** Min Li

**Affiliations:** 1 Taiyuan Normal University, Jinzhong, China

This is a high-quality genomic data report with standardized experimental design, mature technical implementation, and reliable data quality, fully meeting the publication standards for open science and genomics research.

 I suggest only a few minor comments:

1. Were the three sequenced specimens identified by both morphological method and DNA barcoding process? Before DNA extraction, were the specimens undergone aseptic treatment?
2. Clarify the sex of the specimen (specimen ID NHMUK015058720, ToLID icDroQuad2) used for Hi-C sequencing to ensure consistency between assembly results and sex-related chromosome annotations.
3. Supplement key steps of the ASCC decontamination pipeline (e.g., contamination sequence identification criteria, filtering thresholds) to illustrate how the purity of the genome assembly is ensured.
4. If the annotations of the genome are also included, it would be more comprehensive.

Are sufficient details of methods and materials provided to allow replication by others?

Yes

Is the rationale for creating the dataset(s) clearly described?

Yes

Are the datasets clearly presented in a useable and accessible format?

Yes

Are the protocols appropriate and is the work technically sound?

Yes

Reviewer Expertise:

molecular evolutionary biology; molecular ecology; DNA metabarcoding technology

I confirm that I have read this submission and believe that I have an appropriate level of expertise to confirm that it is of an acceptable scientific standard.
